# Genome-Wide Identification, Characterization, and Expression Analysis of *SPIRAL1* Family Genes in Legume Species

**DOI:** 10.3390/ijms24043958

**Published:** 2023-02-16

**Authors:** Qianxia Yu, Junjie Liu, Jiayu Jiang, Fudong Liu, Zhen Zhang, Xiaoye Yu, Mengru Li, Intikhab Alam, Liangfa Ge

**Affiliations:** 1 Department of Grassland Science, College of Forestry and Landscape Architecture, South China Agricultural University, Guangzhou 510642, China; 2 College of Life Sciences, South China Agricultural University, Guangzhou 510642, China; 3 Guangdong Subcenter of the National Center for Soybean Improvement, College of Agriculture, South China Agricultural University, Guangzhou 510642, China; 4 Guangdong Laboratory for Lingnan Modern Agriculture, Guangzhou 510642, China

**Keywords:** legume, *Medicago truncatula*, *Glycine max*, *SPIRAL1*, microtubules, salt stress

## Abstract

The *SPIRAL1* (*SPR1*) gene family encodes microtubule-associated proteins that are essential for the anisotropic growth of plant cells and abiotic stress resistance. Currently, little is known about the characteristics and roles of the gene family outside of *Arabidopsis thaliana*. This study intended to investigate the *SPR1* gene family in legumes. In contrast to that of *A. thaliana*, the gene family has undergone shrinking in the model legume species *Medicago truncatula* and *Glycine max.* While the orthologues of *SPR1* were lost, very few *SPR1-Like* (*SP1L*) genes were identified given the genome size of the two species. Specifically, the *M. truncatula* and *G. max* genomes only harbor two *MtSP1L* and eight *GmSP1L* genes, respectively. Multiple sequence alignment showed that all these members contain conserved N- and C-terminal regions. Phylogenetic analysis clustered the legume SP1L proteins into three clades. The *SP1L* genes showed similar exon-intron organizations and similar architectures in their conserved motifs. Many essential cis-elements are present in the promoter regions of the *MtSP1L* and *GmSP1L* genes associated with growth and development, plant hormones, light, and stress. The expression analysis revealed that clade 1 and clade 2 *SP1L* genes have relatively high expression in all tested tissues in *Medicago* and soybean, suggesting their function in plant growth and development. *MtSP1L-2*, as well as clade 1 and clade 2 *GmSP1L* genes, display a light-dependent expression pattern. The *SP1L* genes in clade 2 (*MtSP1L-2*, *GmSP1L-3*, and *GmSP1L-4*) were significantly induced by sodium chloride treatment, suggesting a potential role in the salt-stress response. Our research provides essential information for the functional studies of *SP1L* genes in legume species in the future.

## 1. Introduction

Microtubules (MTs) are one of the three major cytoskeletal elements in eukaryotic cells. Microtubules are composed of α- and β-tubulin dimers. Typically, 13 protofilaments assemble into tubular microtubule structures [1]. Microtubules perform essential roles in many aspects of a plant cell, including cell division, directional cell expansion, formation of cell walls, and morphogenesis. For instance, cortical MTs, which are anchored to the inner surface of a plasma membrane during interphase, played an important role in defining the final shape of differentiated plant cells by guiding cellulose microfibril deposition and controlling anisotropic cell expansion [2,3,4,5,6,7,8]. Similar to other eukaryotes, the structure of plant MTs is modulated not only by various developmental cues, but also by environmental signals and stress conditions [9,10,11]. Rearrangement of cortical MT arrays has been observed after pathogen attack or exposure to extreme temperature, dehydration, and hyper salinity, indicating the great importance of MTs in biotic and abiotic stresses [12,13,14,15,16,17].

SPIRAL1 (SPR1; At2g03680) is a plant-specific MT-associated protein (MAP) belonging to a six-member family with overlapping functions in *Arabidopsis thaliana* [18,19,20,21]. In *A. thaliana* cells, SPR1 is localized to the MT lattice and partially accumulates at the growing plus ends of MTs, forming an extended comet that is much longer than the END BINDING1 (EB1) comet [19,22]. The *spr1* mutant shows right-handed helical growth in roots and etiolated hypocotyls, and it exhibits an abnormal cortical MT array with a left-handed helical pitch [21]. The six SPR1 family genes in *A. thaliana* share high sequence similarity in the N- and C-terminal regions, and they act redundantly in maintaining anisotropic growth in rapidly elongating cells [18]. In addition to its roles in regulating plant development, SPR1 also plays a key role in the response to abiotic stress-induced MT disassembly [10,11]. SPR1 is degraded by the 26S proteasome, and its degradation rate is accelerated in response to salt stress, thus facilitating MT disassembly and new MT network formation for better survival in high salinity conditions [11]. In addition, SPR1 is also involved in the response to drought stress. SPR1 acts as the phosphorylated substrate of OPEN STOMATA 1 (OST1), facilitates microtubule disassembly in guard cells, and sequentially avoids water loss by controlling stomatal closure [10].

The legume family (Leguminosae) is one of the largest families of Angiosperms, with more than 19,500 species [23,24]. Many legumes are important food crops, providing highly nutritious sources of protein and micronutrients for human beings. Soybean (*Glycine max*) is a worldwide important crop legume that contains significant amounts of protein, oil, and micronutrients. *Medicago truncatula* is a close relative of alfalfa (*M. sativa*), the famous forage legume. Currently, soybean and *M. truncatula* have been used as models for understanding growth and development in legumes.

In this study, the *SPR1* family genes were identified in Leguminosae and analyzed in two species, *M. truncatula* and *G. max*. Multiple sequence alignment, phylogenetic relationships, gene structure, protein motifs, cis-acting elements, protein folding, chromosome location, and collinearity were systematically analyzed. The expression of *SPR1-Likes* (*SP1Ls*) in different organs and tissues was analyzed in *M. truncatula* and *G*. *max* grown in white-light and dark conditions. To explore the role of legume *SP1Ls* in the saline stress response, we also analyzed the expression of *SP1Ls* in response to salt stress in *M. truncatula* and *G*. *max*. We found that clade 1 and clade 2 *SP1Ls* are substantially expressed in all tested tissues of *Medicago* and soybean and display a light-dependent expression pattern. Additionally, clade 2 *SP1Ls* were significantly induced by saline stress.

## 2. Results

### 2.1. Identification and Phylogenetic Analysis of SPIRAL1-like (SP1L) Genes in Legume Species

**Figure 1:**
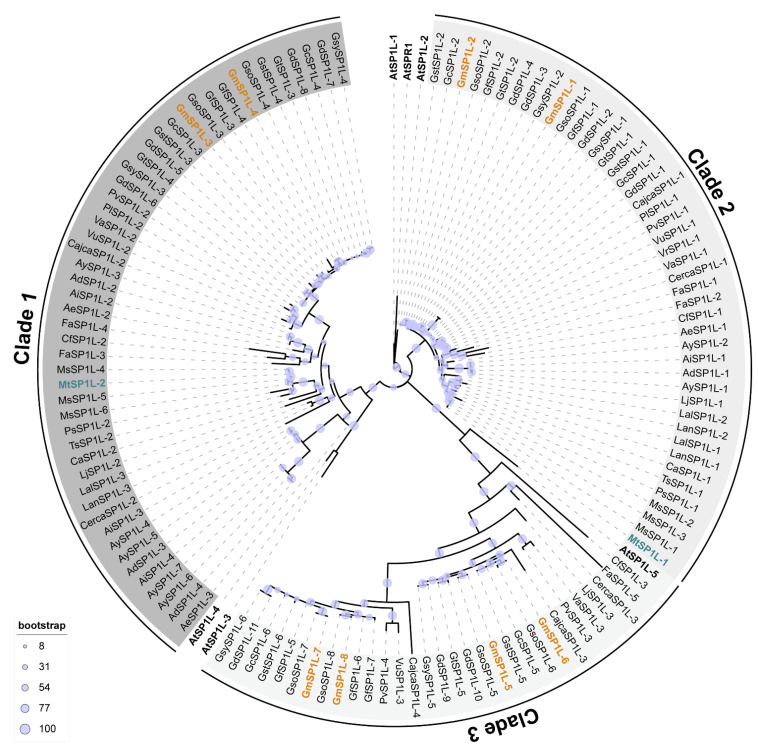
Phylogenetic analysis of the SPIRAL1 (SPR1) families across the Leguminosae species and *Arabidopsis thaliana*. Clades are shaded with different gradients of grey. The *Arabidopsis* (*At*) SPR1 family proteins are bold, and the SPR1-Like (SP1L) proteins from *Medicago truncatula* (*Mt*) and *Glycine max* (*Gm*) are highlighted with blue and orange, respectively.

A total of 126 SP1L-related sequences were obtained from 29 species of Leguminosae. The characteristics of the identified proteins, including chromosome location, CDS and protein length, molecular weight (MW), and isoelectric point (pI), are listed in Supplementary Table S1. Based on the current genome annotation, the number of SP1L members ranged from one (*Vigna radiata*) to eleven (*G. dolichocarpa*). Protein sequence lengths ranged from 69 (Glydo.01G000671) to 177 (Phalu.04G0000041500) amino acids, corresponding to MWs of between 6.60 kDa and 18.34 kDa, with an average of 11.09 kDa. The pIs varied from 4.47 (Aradu_6Y48C, Arahy_J0K3K7, Arahy_W61CSA, and Araip_5XB02) to 9.78 (Cerca_209S17695). All the data suggested a high variability among *SP1L* genes in the legume species genomes. Sequence-based phylogenetic analysis showed that the Leguminosae SP1L proteins were grouped into three distinct clusters (Figure 1). The two SP1Ls in *M. truncatula* are grouped in clade 1 (MtSP1L-1) and clade 2 (MtSP1L-2), respectively. *G. max* has eight SP1L candidates which are distributed in all three clades as follows: GmSP1L-1 and GmSP1L-2 belong to clade 1, GmSP1L-3 and GmSP1L-4 belong to clade 2, and GmSP1L-5 to GmSP1L -8 belong to clade 3. The length of the predicted proteins of MtSP1Ls and GmSP1Ls varied from 104 to 139 aa and 87 to 130 aa, respectively. The pI values of the two MtSP1Ls were 9.16 and 9.26, and they displayed a wider range in soybean (Table 1). The corresponding MWs of MtSP1Ls and GmSP1Ls ranged from 1.01 to 1.36 and from 0.92 to 1.24 kDa, respectively.

In order to clarify the orthologous relationship among *AtSP1Ls*, *MtSP1Ls*, and *GmSP1Ls*, we performed maximum likelihood (ML) phylogenetic analyses for the *SP1L* genes from *A. thaliana*, *M. truncatula*, and *G. max* (Supplementary Figure S1). Consistent with the phylogenetic relationships illustrated in Figure 1, all the legume *SP1Ls* were grouped into three clusters. With the bootstrap support value of 0.945, *AtSP1L-5* of *A. thaliana* was grouped into clade 3, which also contained *GmSP1L-5*, *GmSP1L-6*, *GmSP1L-7*, and *GmSP1L-8*. However, the other five *AtSP1L* genes were located relatively far from the legume *SP1Ls* in the phylogenetic distance and were not able to cluster with the legume genes (Figure 1 and Supplementary Figure S1).

### 2.2. Multiple Sequence Alignment of SP1L Genes in Medicago, Soybean, and Arabidopsis

**Figure 2:**
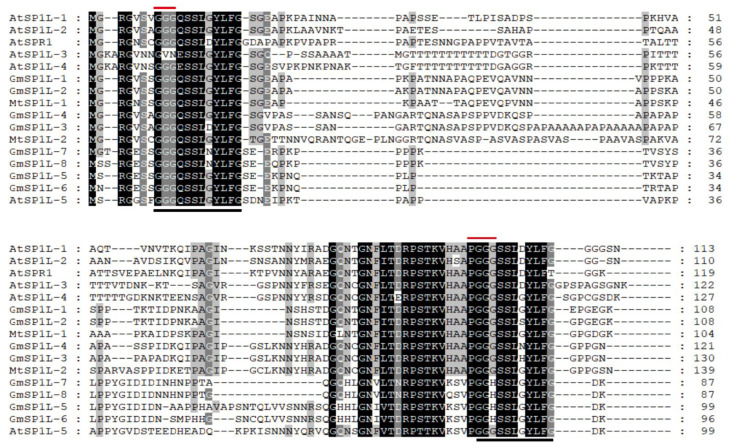
Protein sequences alignment. The identical amino acid residues among all proteins are shown in black boxes, and the conserved residues are shown in gray boxes. The two solid lines under the sequences mark the locations of a direct repeat sequence. The predicted MT binding motifs (GGG and PGGG) are marked with red lines.

To further investigate the conserved regions of the SP1L proteins, multiple protein sequence alignments were conducted. The SP1L proteins in *M. truncatula* and *G. max* shared high sequence similarity with the AtSP1Ls in the N- and C-terminal regions, whereas the central sequences were poorly conserved (Figure 2). Further, a highly conserved direct repeat sequence was present at the N-terminal and C-terminal ends of the MtSP1L and GmSP1L proteins, with the consensus motif being GG(G/H)(Q/-)SSL(G/D/N/S/H)YLFG (Figure 2, underlined with solid lines). At the N-terminal and C-terminal of these two direct repeat sequences, a Gly-Gly-Gly (GGG) motif and a Pro-Gly-Gly-Gly (PGGG) motif were present, and these are likely responsible for MT binding in many mammalian MAPs [20,25]. These results indicate that a similar pattern of conservation exists between the Brassicaceae SP1L and Leguminosae homologs.

The secondary structures of the AtSP1L, MtSP1L, and GmSP1L proteins were predicted. The results showed that the SPR1L genes in these three species all showed very simple 2D structures (Supplementary Table S2). Only AtSPR1, AtSPR5, MtSP1L-1, and GmSP1L-4 had alpha helices, ranging from 3.31–8.4%. The extended strands found in AtSP1L-3 (16.39%) and AtSP1L-4 (14.17%) were absent in the MtSP1L and GmSP1L proteins. The 3_10_-helices were found in some SP1L proteins of *A. thaliana* and *G. max* (AtSP1l-2, AtSP1L-4, AtSP1L-5, and GmSP1L-1), ranging from 2.36–4.04%, but they were absent in the MtSP1Ls. Other than that, the rest of the regions took up a great proportion of the SP1L proteins, which were composed of turns (8.05–22.13%), bend regions (8.2–16.09%), and other states (53.28–77.08%). Furthermore, the 3D structures of the six AtSP1L, two MtSP1L, and eight GmSP1L proteins were predicted using AlphaFold2 [26], and they are shown in Supplementary Figure S2. The five obtained AlphaFold models of each SP1L protein displayed a similar folding pattern. These results show that genes in the same clades share a great similarity in terms of protein configuration, which implies a similar functions of these proteins.

### 2.3. Analysis of Gene Structure and Conserved Motifs in SP1L Genes

**Figure 3:**
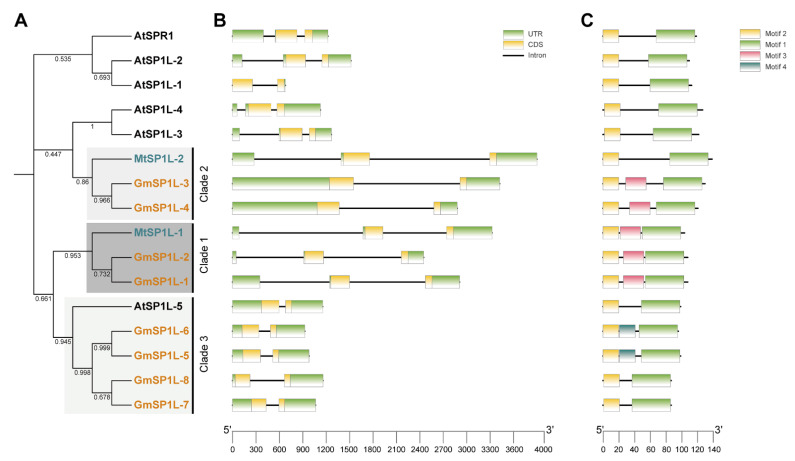
Phylogenetic relationships, gene structures, and motifs of the *SP1L* genes from *A. thaliana*, *M. truncatula,* and *G. max* (**A**–**C**). The clades and colors of the phylogenetic tree are the same as in Figure 1. The green boxes in (**B**) indicate 5′- and 3′- untranslated regions, the yellow boxes indicate exons, and the black lines indicate introns. The motifs are indicated in different colored boxes with different numbers, and the sequence information for each motif is provided in Supplementary Figure S3.

We next analyzed the gene structure and conserved motifs for the identified *SP1L* genes. As shown in Figure 3, the genes of the *MtSP1Ls* and *GmSP1Ls* all had one intron and two exons, which was consistent with those in the *AtSP1Ls* (Figure 3B). These results demonstrated that the *AtSP1Ls*, *MtSP1Ls*, and *GmSP1Ls* exhibited conserved intron/exon structures.

Five conserved motifs in the SP1L proteins of *A. thaliana*, *M. truncatula,* and *G. max* were identified (Supplementary Figure S3), and their positions are shown in Figure 3C. Most of them had similar motif positions and types. All SP1L members contained motifs 1 and 2. Three members of clade 1 and GmSP1L-3 and GmSP1L-4 in clade 2 contained an additional motif 3, whereas GmSP1L-5 and GmSP1L-6 in clade 3 contained motif 4. The proteins that shared similar motif compositions are likely to have the same gene functions.

### 2.4. Analyses of the Chromosomal Distribution and Synteny in the SP1L Genes in Medicago and Soybean

**Figure 4:**
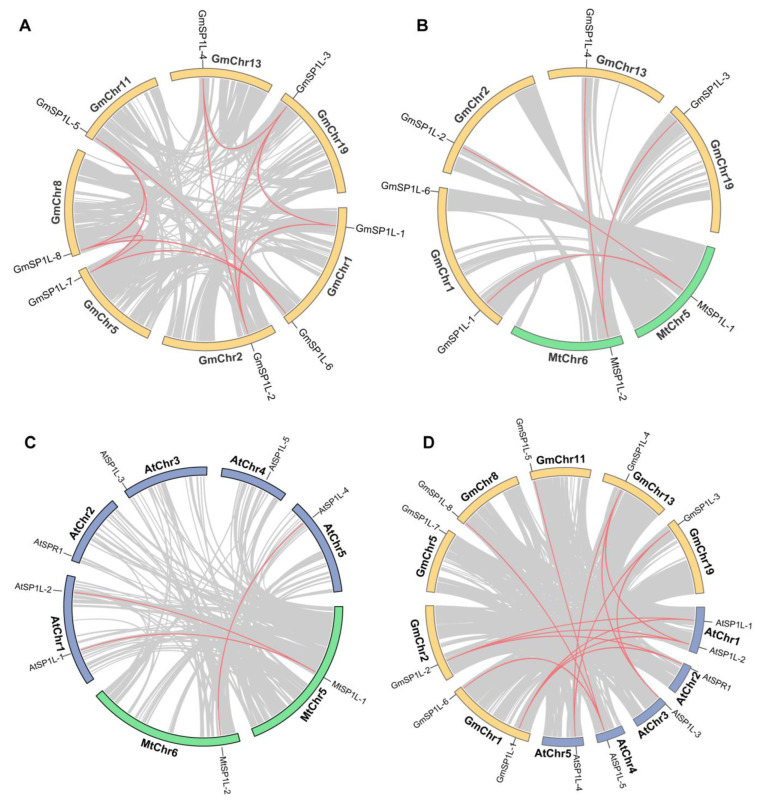
Chromosome distributions of the *SP1Ls* in *M. truncatula* and *G. max*. (**A**) The chromosomal location and interchromosomal relationship of the *GmSP1Ls* in *G. max*. The segmentally duplicated genes are connected by red lines. Synteny analysis of the *SP1L* genes between (**B**) *M. truncatula* and *G. max*; (**C**) *M. truncatula* and *A. thaliana*; and (**D**) *G. max* and *A. thaliana*. The gray lines in the background indicate the collinear blocks and the red lines highlight the syntenic *SP1L* gene pairs.

As illustrated in Figure 4A, eight *GmSP1L* genes were disproportionately distributed across the seven chromosomes of *G. max*. *GmSP1L-1* and *GmSP1L-6* were distributed on the same chromosome (Chr1), while other homologous genes were distributed on six different chromosomes (Chr2, 5, 8, 11, 13, and 19). In *M. truncatula*, the two *SP1L* homologous genes were distributed on Chr5 and Chr6, respectively (Figure 4B).

Syntenic blocks within the *G. max* genome were examined to identify relationships among the *GmSP1L* genes and potential gene duplication events. Eleven gene pairs were found in the *G. max* genome, and they were located on different chromosomes (Figure 4A), indicating that segmental duplications in these regions likely contributed to the expansion of the *GmSP1L* family.

Three comparative syntenic maps of the *SP1L* genes were constructed at the genome-wide level across the three species (*M. truncatula*, *G. max*, and *A. thaliana)* (Figure 4B–D). Four syntenic gene pairs were identified between *M. truncatula* and *G. max*: *MtSP1L-1* is the orthologous gene of *GmSP1L-1* and *GmSP1L-2*, and *MtSP1L-2* is the orthologous gene of *GmSP1L-3* and *GmSP1L-4* (Figure 4B). These orthologous pairs may have existed before the ancestral divergence. A total of only three syntenic gene pairs were identified between *Arabidopsis* and *M. truncatula*: *MtSP1L-1* is homologous to *AtSP1L-1* and *AtSP1L-2*, while *MtSP1L-2* is homologous to *AtSP1L-4* (Figure 4C). In addition, 14 pairs of syntenic genes were found between the genome of *G. max* and *A. thaliana,* as shown in Figure 4D.

### 2.5. Analysis of the Cis-Elements in the Promoter Sequences of the SP1L Genes in Medicago and Soybean

**Figure 5:**
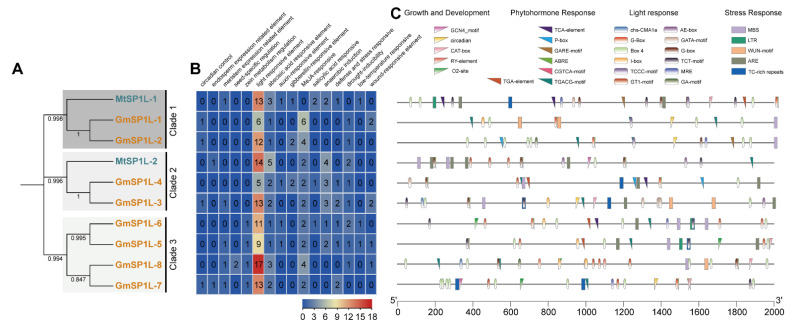
Putative cis-elements and transcription factor binding sites in the promoter regions of the *SP1L* genes from *M. truncatula* and *G. max*. (**A**) The groups and color are as indicated in Figure 1. (**B**) The color and number of the grid indicate the numbers of different cis-acting elements in these *SP1L* genes. (**C**) The colored blocks represent different types of cis-acting elements and their locations in each *SP1L* gene.

The cis-acting elements are important for the binding of transcription factors, which control the expression of their downstream target genes. The promoter sequences of 2000 bp for the two *MtSP1L* and eight *GmSP1L* genes were analyzed. A total of 231 putative cis-acting elements were identified in the promoter regions of these *SP1L* genes (Supplementary Table S3). Overall, the promoters of the *MtSP1L* and *GmSP1L* genes contained various cis-acting elements with different numbers. All these identified cis-elements were classified into four groups based on their participation in various biological processes: plant growth and development (17), phytohormone responsive (60), light-responsive (113), and stress-responsive (41) (Figure 5 and Supplementary Table S3). Compared to the other three groups, light-responsive-related cis-acting elements (i.e., G-box, Box 4, and the TCT motif) were found at a very high frequency in all *Mt* and *Gm SP1L* genes (Figure 5B), indicating that these genes’ expression and function may be light-dependent. In addition, several stress-related promoter regions were also identified in the *Mt* and *Gm SP1L* genes, including anaerobic induction (15), wound induction (5), drought (10), low-temperature (3), and defense (8). Moreover, the primary factors related to growth and development included meristem-related (5), seed-related (2), circadian-related (4), zein metabolism-related (4), and endosperm expression (2) cis-acting elements, and the hormone-related elements included methyl jasmonate (MeJA) (26), abscisic acid (ABA) (21), ethylene (81), gibberellin (GA) (5), salicylic acid (SA) (4), and auxin (4) -responsive regulators, which were also identified in the *MtSP1Ls* and *GmSP1Ls* promoters. These results suggest that various cis-acting promoter elements may regulate the expression of the *MtSP1L* and *GmSP1L* genes during plant growth and stress responses.

### 2.6. Expression Patterns of SP1L Genes in Various Tissues of M. truncatula and G. max

**Figure 6:**
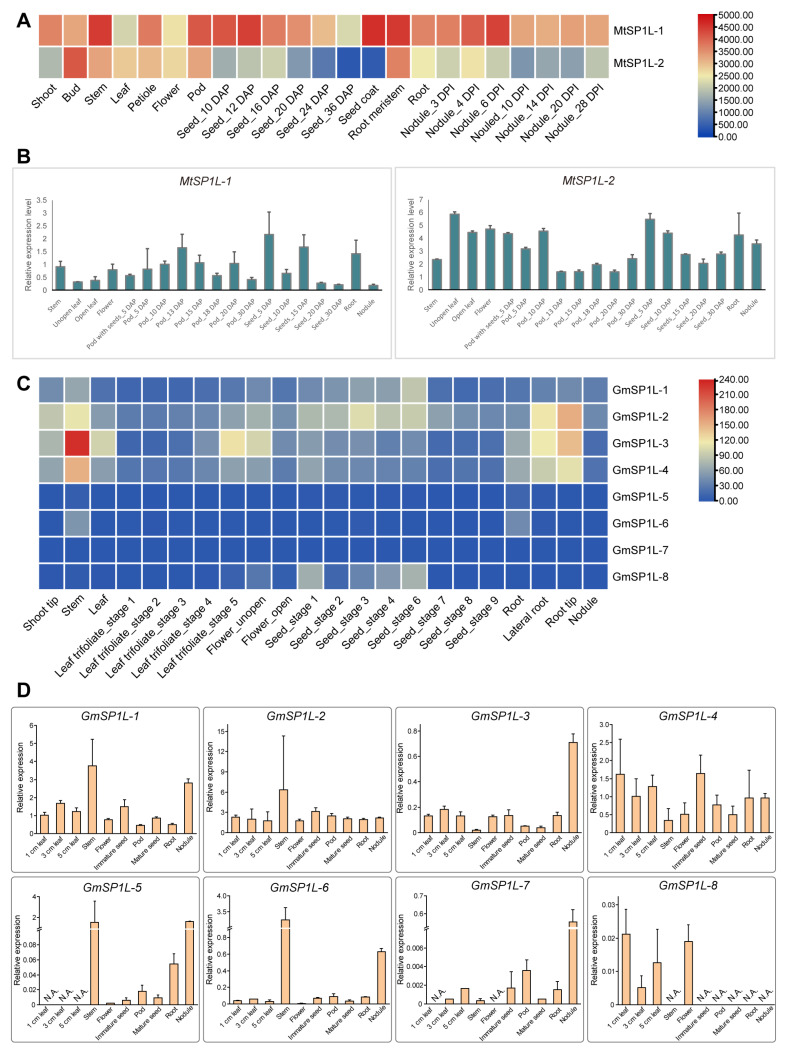
Tissue expression profiles of the *SP1L*s in *Medicago* and soybean. (**A**) The expression profiles of two *MtSP1L* genes in different tissues retrieved from the GeneChip dataset. (**B**) The expression levels of the *MtSP1L* genes in various tissues verified by qRT-PCR. (**C**) The transcriptional levels of eight *GmSP1L* genes in different tissues of soybean were analyzed using the public data in Phytozome. (**D**) The expression levels of the *GmSP1L* genes in various tissues of Williams 82 verified by qRT-PCR. The color scale shows increasing expression levels from blue to red in (**A**,**C**). The qRT-PCR results are expressed as the ratio of *SP1Ls* expression normalized against the expression of the reference genes. All data represent the means ± SDs of three biological replicates. ‘N.A.’ indicates undetectable expression.

To understand the expression patterns of the *SP1L* genes in various tissues of *M. truncatula*, the expression profiles in the GeneChip dataset were investigated. It was shown that both of the *MtSP1L* genes displayed specific expression in all tested tissues or organs in various developmental stages, including shoots, vegetative buds, stems, leaves, flowers, pods, seeds, roots, and nodules (Figure 6A). Furthermore, the qRT-PCR-based tissue-specific transcript abundance analysis of seven tissues (stems, leaves, flowers, pods, seeds, roots, and nodules) with different stages was conducted with gene-specific primers, and the transcript expression levels of two *Medicago SP1L* genes are shown in Figure 6B. Both transcripts of *MtSP1L-1* and *MtSP1L-2* were detected in all organs, suggesting their potential roles in plant growth and development in *Medicago*.

In soybean, the data from Phytozome showed that clade 1 (*GmSP1L-1* and *GmSP1L-2*) and clade 2 (*GmSP1L-3* and *GmSP1L-4*) genes had relatively higher expression levels and were detected in all tested tissues; however, clade 3 *GmSP1L*s (*GmSP1L-5*, *GmSP1L-6*, *GmSP1L-7*, and *GmSP1L-8*) showed a relatively lower expression (Figure 6C). The results of the qRT-PCR show that the clade 1 and clade 2 genes had clear differential expression in different organs, and some of them exhibited highly tissue-specific expression, while the expression levels of the clade 3 *GmSP1L*s were too low to be detected in most of the tissues, but they were selectively expressed in particular tissues or organs (Figure 6D). The results of the qRT-PCR were generally consistent with the published data, with some exceptions, for example, in the qRT-PCR data, the maximum expressions of the *GmSP1L-1* gene were in stems, *GmSP1L-3* were in nodules, *GmSP1L-5* and *GmSP1L-6* were in stems and nodules, and *GmSP1L-7* were in nodules; while in the Phytozome data, the maximum expressions of the *GmSP1L-1* gene were in the seeds of stage 6 and for *GmSP1L-3* in the stems, and *GmSP1L-5* and *GmSP1L-7* showed no expression in any tissues. These differences may have resulted from the different timing of the sampling.

In conclusion, the results indicated that the clade 1 and clade 2 *SP1L* genes in *M. truncatula* and *G. max* may play a significant role in plant growth, while the clade 3 gene may be redundant and less important in organ development in legumes.

### 2.7. Comparison of SP1Ls’ Expression Patterns in the Light- and Dark-Grown Hypocotyls in M. truncatula and G. max

**Figure 7:**
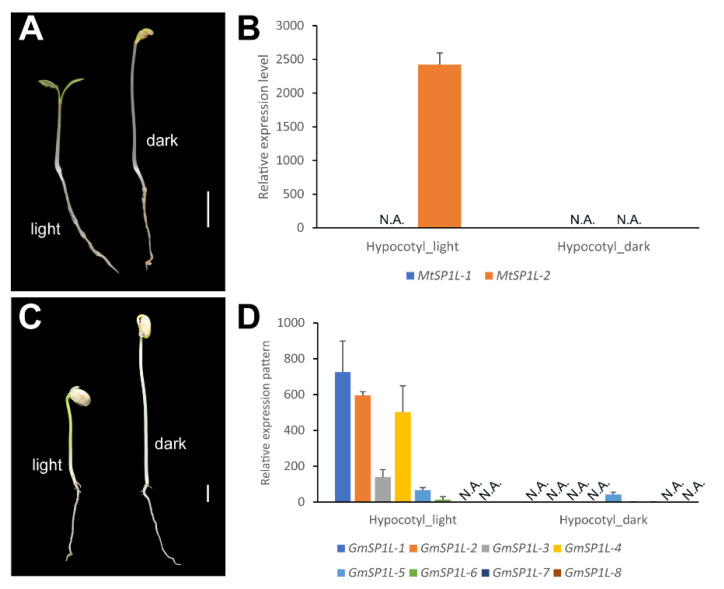
The expression patterns of *SP1L* genes in response to light stress in *M. truncatula* (R108) and *G. max* (Williams 82). The seedlings of (**A**) *M. truncatula* and (**C**) *G. max* under white-light and dark growth conditions. The qRT-PCR shows the relative expression levels of the *SP1L* genes in (**B**) *M. truncatula* and (**D**) *G. max* hypocotyls. The bar in (**A**,**C**) equals 1 cm. ‘N.A.’ indicates undetectable expression.

To investigate the gene expression patterns in response to light, we performed a comparative expression analysis for the *SP1L*s in the hypocotyls of seedlings under white light and dark conditions (Figure 7). In both *M. truncatula* and *G. max*, hypocotyls of the dark-grown seedlings elongated rapidly after five days of growth, and they were much longer than those grown in the white light on the fifth day (Figure 7A,C). This was coincident with the phenomenon in *Arabidopsis*, which has been reported before [27]. In *M. truncatula*, the transcript level of *MtSP1L-1* was not influenced in response to light, as they were not detected in hypocotyls in either the light or dark conditions, while *MtSP1L-2* displayed a significantly higher expression level in white light than in darkness (Figure 7B). In *G. max*, the *SP1L* genes in clade 1 and clade 2 were expressed differentially under different light conditions. Specifically, the expression levels of clade 1 and 2 *GmSP1L*s in the light-grown hypocotyls were higher than those of the dark-grown hypocotyls. In contrast, the clade 3 *GmSP1L* genes (*GmSP1L-5*, *GmSP1L-6*, *GmSP1L-7,* and *GmSP1L-8*) showed no response to light, and they displayed relatively lower expression levels in the hypocotyls grown in both light and dark conditions (Figure 7D). These results indicated that the expression levels of *MtSP1L-2* in *Medicago*, as well as those of *GmSP1L-1*, *GmSP1L-2*, *GmSP1L-3,* and *GmSP1L-4* in soybean, are light-dependent in the respective hypocotyls.

### 2.8. The Expression of SP1Ls under Saline Stress

**Figure 8:**
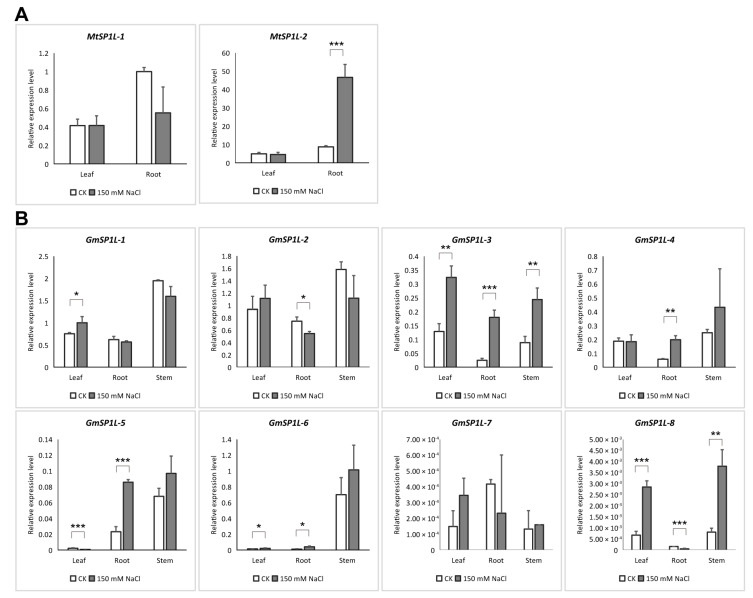
Effects of salt treatments on the *SP1L*s transcript levels in *M. truncatula* and *G. max*. The expression levels of the *MtSP1Ls* (**A**) and *GmSP1Ls* (**B**) genes under sodium chloride treatments. The asterisk indicates significant differences among mean values compared with the control group (Student’s *t*-test: *** *p* < 0.001, ** 0.001 < *p* < 0.01, and * 0.01 < *p* < 0.1). The results were based on three replicates in three independent experiments.

The expression levels of the *MtSP1L* and *GmSP1L* genes were analyzed under sodium chloride treatments by qRT-PCR analysis. Most *SP1L*s were induced at different levels (Figure 8). The expression level of *MtSP1L-2* was induced more than five-fold in the roots after sodium chloride treatment (Figure 8A). The orthologous genes of *MtSP1L-2* in soybean, *GmSP1L-3*, and *GmSP1L-4* had a similar salt-induced expression pattern. The expression level of *GmSP1L-3* was induced by more than 2.5- to 7-fold in leaves, roots, and stems after a sodium chloride treatment, and the expression level of *GmSP1L-4* was induced by more than three-fold in roots after a sodium chloride treatment (Figure 8B). Moreover, the expression levels of the *GmSP1L-1* genes were transiently elevated under a sodium chloride treatment, while the expression levels of *MtSP1L-1* and *GmSP1L-2* were reduced after a sodium chloride treatment. The overall expression levels of clade 3 genes were relatively low, which is consistent with the results shown above (Figure 6D, Figure 7D and Figure 8B). Overall, the results revealed that the *SP1L* genes in clade 2 were likely to be involved in salt stress responses in legume species.

## 3. Discussion

During genome evolution, gene duplication and gene loss events can contribute to the contraction and expansion of gene families. Genome changes can be linked to evolutionary processes that result in environmental niche adaptation [28]. Contrary to the common view of a larger gene family size in legumes than in *Arabidopsis* [29,30,31,32], the BLASTp and phylogenetic analyses in this study revealed that the *SPR1* gene family in many legume species is smaller than its counterpart in *Arabidopsis* (Figure 1 and Supplementary Table S1), indicating the contraction of the *SPR1* gene family in legume genomes. In addition, *SPR1*, which plays a significant role in plant growth and abiotic stress in *Arabidopsis*, was absent in the *M. truncatula* and *G. max* genomes. Currently, it is unclear why *SPR1* has been lost in legumes, though it may have been caused by changes in the living environment such that this gene is no longer essential for the plants’ fitness [28]. Additionally, other *SP1Ls* may have replaced the function of *SPR1* in legumes.

Many MAPs, including the *SPR1* family, are likely to have functional redundancy among members of their multigene families. In *Arabidopsis*, the overexpression of each *SP1L* rescued the helical growth phenotype of *spr1*, indicating that the six members of SPR1 family proteins share the same biochemical functions in maintaining the cortical MT organization essential for anisotropic cell growth [18]. In Leguminosae, the number of putative *SP1L* genes varies from one (*V. radiata*) to eleven (*G. dolichocarpa*) (Figure 1 and Supplementary Table S1). The soybean (*G. max*) genome is known to have a high degree of redundancy as a result of both whole genome duplication and tandem gene arrays. Up to eight *GmSP1L* members are identified in *G. max,* while there are only two *MtSP1L* genes in the *M. truncatula* genome. It is interesting to note that clade 3 *SP1L* genes are lacking in both the *M. truncatula* and *M. sative* genomes (Figure 1). According to the expression pattern analysis, the *GmSP1L* genes in clade 3, including *GmSP1L-5*, *GmSP1L-6*, *GmSP1L-7,* and *GmSP1L-8*, are rarely expressed in most organs or tissues (Figure 6C,D). Moreover, their expression levels are nearly undetectable in etiolated hypocotyls (Figure 7D), and they were not induced by salt stress (Figure 8B), suggesting that the clade 3 *SP1L* genes are less important in legume species. In addition, it is likely that the eight *GmSP1Ls* and two *MtSP1Ls* function redundantly in plant development and growth, especially in members of the same clade which evolved from recent common ancestors and show similar gene structures and expression patterns.

Photomorphogenesis is light-mediated development through which plants are able to develop growth patterns in response to the light spectrum [33]. For plants germinating in soil, hypocotyl elongation is essential for initiating autotrophic growth by exposing them to light [27]. Previous studies have shown that in rapidly elongating cells of etiolated hypocotyls, more SPR1 proteins are required to align the cortical MT arrays transversely when the cells expand vertically [18]. Thus, in *Arabidopsis*, the helical hypocotyls of *spr1* mutant are found in dark-grown seedlings but not in light-grown seedlings [20]. The function of the *SP1L* gene in light-regulated cell expansion has been confirmed in *Salix matsudana* (Salicaceae) [34]. SPR1 in *S. matsudana* (SmSPR1) interacts with the COP9 signalosome subunit 5A (CSN5A) and ELONGATED HYPOCOTYL 5 (HY5), further demonstrating the involvement of the *SP1L* genes in light-regulated pathways [34]. In this study, we found that the *SP1L* genes in *M. truncatula* and *G. max* had an extremely high frequency of light-responsive-related cis-acting elements in the promoter regions (Figure 5B), which suggests that these genes may be light-dependently expressed. *MtSP1L-2* and *GmSP1L-1* to *-4* expression levels are much higher in light-grown hypocotyls than in dark-grown hypocotyls (Figure 7B,D). Though this finding is the opposite of *SPR1* in *Arabidopsis* [18], it is consistent with *AtSP1L-2*, which was found in light-grown hypocotyls but not in dark-grown seedlings [18]. The differences in expression patterns may result from some special regulatory elements, modifications in their promoters, or functional segregation during evolution. As stabilized MTs are essential for maintaining the shape of cells, the light-dependent expression of *SP1Ls* genes may be relevant to the inhibition of hypocotyl elongation by light.

Salinity is a serious threat to agriculture, affecting plant growth and crop yields [35]. In legumes, rhizobia are known to be very sensitive to salinity. Thus, symbiotic interaction will be largely affected by salt stress, leading to a reduction in nodule number and limited nitrogen fixation [36,37]. In *Medicago*, short-term salinity stress was shown to affect the realignment of MTs from transverse to parallel [38], suggesting the involvement of MTs in the salt-stress response. The mechanisms by which SPR1 is degraded by the 26S proteasome in response to salt stress, which then induces disassembly of the cortical microtubules, have been well-studied in *Arabidopsis*. It has been shown that SPR1 depletion in response to salt is, indeed, the result of a posttranscriptional mechanism, and the *SPR1* mRNA level did not change in response to salt stress treatments [11]. In contrast, in legume species, a significant induction of clade 2 *SP1L* genes was detected after sodium chloride treatments (Figure 7). These results indicate that the *SP1L* genes from legumes may play a different role in response to saline stress, which is interesting and should be investigated in the future.

## 4. Materials and Methods

### 4.1. Identification of the SP1L Proteins in Legume Genomes

To identify SP1L proteins in legume species, we conducted BLASTp searches against the Legume genome database LIS (the Legume Information System, https://www.legumeinfo.org/, accessed on 21 May 2022) using previously reported *Arabidopsis* SPR1 and SP1L proteins as query sequences. The ProtParam web tool (SIB Swiss Institute of Bioinformatics, Lausanne, Switzerland, http://web.expasy.org/protparam/, accessed on 15 June 2022) was used to determine the molecular weights (Wts) and theoretical isoelectric points (pIs) of the identified SP1L proteins.

### 4.2. Phylogenetic Analysis and Multiple Sequence Alignment

The phylogenetic tree of the SP1Ls in legumes was constructed using PhyloSuite v1.2.2 [39]. In PhyloSuite, the full-length protein sequences of all AtSP1Ls and SP1Ls from legume species were aligned with MAFFT [40]. The optimal model JTT+R3 was calculated by Modelfinder [41] based on the Bayesian information criterion (BIC) standard. ML phylogenies were inferred using IQ-TREE [42] under the JTT+R3 model with 10,000 ultrafast bootstraps [43]. The ML phylogenetic analysis for the SP1Ls from *A. thaliana*, *M. truncatula,* and *G. max* was conducted using MEGA-X [44] with 1000 standard bootstrap replicates. The online software iTOL v6.6 (https://itol.embl.de/, accessed on 29 June 2022) was used to modify the phylogenetic trees [45]. Multiple protein sequence alignments of the SP1Ls were analyzed using ClustalX2 [46] and displayed via GeneDoc [47].

### 4.3. Gene Structure and Motif Analysis

The conserved motifs were identified by selecting motifs from the MEME program v5.1.0 (Multiple Expectation Maximization for Motif Elicitation, University of Nevada, Reno and University of Washington, USA, http://meme-suite.org/tools/meme, accessed on 22 October 2022), with the motif number set as 20 and the width range of 10 to 200 amino acids (aa). The exon-intron structures were retrieved from the gene annotation files. The visualizations of the exon–intron positions and conserved motifs were executed through the TBtools software [48], and the color was adjusted with Adobe Illustrator.

### 4.4. 3D Structural Analysis of the SP1L Proteins

The secondary structures were predicted with the SOPMA model. Protein folding analysis was carried out using Alphafold2 on the Google Colab platform (https://colab.research.google.com/github/sokrypton/ColabFold/blob/main/AlphaFold2.ipynb, accessed on 5 November 2022) [26]. UCSF ChimeraX was used to display the 3D structures of the SP1L proteins [49].

### 4.5. Analyses of the Chromosome Locations and Collinearity of the AtSP1L, MtSP1L, and GmSP1L Genes

The chromosome locations of the *SP1L* genes were obtained from the genome annotation data. The Multiple Collinear Scan Toolkit (MCScanX) was used to analyze the gene duplication events using default parameters [50]. The intraspecific synteny relationship (*M. truncatula* and *G. max*) and interspecific synteny relationships (*M. truncatula* and *G. max*, *M. truncatula* and *A. thaliana*, and *G. max* and *A. thaliana*) were analyzed.

### 4.6. Cis-Acting Elements Analysis

The promoter sequences (2 kb upstream of the translation start site) of the *MtSP1L* and *GmSP1L* genes were identified using the TBtools software [48], and the cis-elements in the promoters regions were predicted with the online program PlantCARE (http://bioinformatics.psb.ugent.be/webtools/plantcare/html/, accessed on 13 October 2022) [51]. TBtools was used to visualize the cis-acting elements of all the *SP1L* genes of *M. truncatula* and *G. max* [48].

### 4.7. Plant Materials

The M. truncatula (R108 ecotype) and G. max (Williams 82 and Guizao 1) plants used in this study were stored in the Department of Grassland Science, College of Forestry and Landscape Architecture, South China Agricultural University. The plants were grown in a growth chamber at 25 °C under a photoperiod of 16 h/8 h in a light/dark regime (80 µmol photons m^−2^s^−1^) and 80–90% humidity. Stems, leaves, flowers, pods, seeds, roots, and nodules of the mature M. truncatula (R108) and G. max (Williams 82) plants were collected separately. All samples were frozen in liquid nitrogen once collected and stored at −80 °C for subsequent RNA extraction and qRT-PCR analysis. To investigate the expression patterns of the SP1L genes in response to light, the seeds (R108 and Williams 82) were germinated under white light or dark conditions. When the cotyledons extended, the hypocotyls of each treatment were collected. The salt stress assays were carried out following the protocol in the *Medicago truncatula* handbook [52]. The germinated seeds (R108 and Guizao 1) were transferred to pots with a 3:1 mix of perlite and sand. The seedlings were irrigated with Hoagland solution. After 10 days of growth, the treatment group was irrigated with a 150 mM sodium chloride solution and then, 24 h later, the plant materials of M. truncatula (R108; leaves and roots) and G. max (Guizao 1; stems, leaves, and roots) were collected for subsequent analysis.

### 4.8. Analysis of the Expression Levels of the SP1L Genes in the Different Organs and Tissues of M. truncatula and G. max

The GeneChip data of two MtSP1L genes were downloaded from the M. truncatula Gene Expression Atlas (https://lipm-browsers.toulouse.inra.fr/pub/expressionAtlas/app/mtgeav3/, accessed on 11 November 2022) [53,54]. The FPKM of eight GmSP1Ls in different tissues were downloaded from Phytozome v13 (https://phytozome-next.jgi.doe.gov/, accessed on 22 November 2022). Heatmaps were generated using TBtools [48]. For the qRT-PCR analysis, the total RNAs were extracted using the Total RNA Extraction Reagent (Vazyme, Nanjing, China) according to the manufacturer’s instructions. First-strand cDNA synthesis was performed using EasyScript One-Step gDNA Removal and a cDNA Synthesis SuperMix kit (TransGen Biotech, Beijing, China). The qRT-PCRs were carried out using a 2×ChamQ Universal SYBR qPCR Master Mix (Vazyme, Nanjing, China) on a CFX96 Real-Time PCR Detection System (Bio-Rad, Hercules, CA, USA). MtACTIN (Medtr3g095530), and GmACTIN (Glyma.18G290800) was used as the endogenous control, respectively. The reaction was carried out as follows: 94 °C for 30 s, followed by 40 cycles of 5 s at 94 °C and 34 s at 60 °C. The relative expression levels of the genes were determined using the comparative 2^−∆∆Ct^ method [55]. Error bars represented the standard deviation. The primer sequences used in this study are shown in Table S4.

## 5. Conclusions

This study analyzed the *SPR1* gene family on a genome-wide scale in legume species. In contrast to *Arabidopsis*, this gene family has undergone shrinking in the two model legume species *M. truncatula* and *G. max*. The orthologues of *SPR1* were lost, and very few *SP1L* genes were identified given the genome size of the two species. These genes show high similarity in the N- and C-terminal regions, whereas the central sequences are poorly conserved. The phylogenetic analysis grouped the SP1L proteins into three clades, and the genes in the same clades share a great similarity in gene structures, protein motifs, cis-acting elements, and protein folding. The *SP1L* genes in clade 1 and clade 2 were highly expressed in all organs and tissues, while the clade 3 *SP1Ls* showed relatively lower expression levels in the examined tissues. Moreover, we found that the *SP1L* genes are sensitive to light conditions or salt-stress in *M. truncatula* and *G. max*. To conclude, this study has provided essential information for further functional studies of *SP1L* genes in legume species.

## Figures and Tables

**Table 1:** Properties of the predicted *SP1L* genes in *M. truncatula* and *G. max*.

Gene Name	TIGR Locus	Chr	Start Site	End Site	Strand	CDS (bp)	Size (aa)	MWs (Da)	pI
*MtSP1L-1*	MtrunA17_Chr5g0419471	Medtr_chr05	19,809,242	19,812,567	-	315	104	10,102.97	9.16
*MtSP1L-2*	MtrunA17_Chr6g0454801	Medtr_chr06	5,010,040	5,013,939	+	420	139	13,634.85	9.26
*GmSP1L-1*	Glyma.01G060900	Glyma_chr01	8,375,261	8,378,172	+	327	108	10,681.55	8.08
*GmSP1L-2*	Glyma.02G119200	Glyma_chr02	11,459,648	11,462,099	+	327	108	10,575.38	9.16
*GmSP1L-3*	Glyma.19G032000	Glyma_chr19	4,032,554	4,035,979	+	393	130	12,443.56	9.36
*GmSP1L-4*	Glyma.13G055400	Glyma_chr13	14,317,887	14,320,766	-	366	121	11,834.82	9.65
*GmSP1L-5*	Glyma.11G021900	Glyma_chr11	1,550,669	1,551,650	-	300	99	10,278.25	8.11
*GmSP1L-6*	Glyma.01G221900	Glyma_chr01	56,191,552	56,192,480	+	291	96	10,170.11	7.97
*GmSP1L-7*	Glyma.05G210800	Glyma_chr05	39,307,017	39,308,083	+	264	87	9177.07	9.10
*GmSP1L-8*	Glyma.08G017200	Glyma_chr08	1,395,764	1,396,925	+	264	87	9161.93	6.82

Note: strand, (-) means antisense strand of chromosome; (+) means positive-sense strand of chromosome; pI, isoelectric point; Mw, molecular weight.

## Data Availability

Not applicable.

## Supplementary Materials

The following supporting information can be downloaded at: https://www.mdpi.com/article/10.3390/ijms24043958/s1.
