# Supplementary figures and images for: Genome-Wide Identification, Characterization, and Expression Analysis of SPIRAL1 Family Genes in Legume Species
Source: Int J Mol Sci. 2023 Feb 16;24(4):3958. doi: 10.3390/ijms24043958 (PMC9959322; doi:10.3390/ijms24043958)

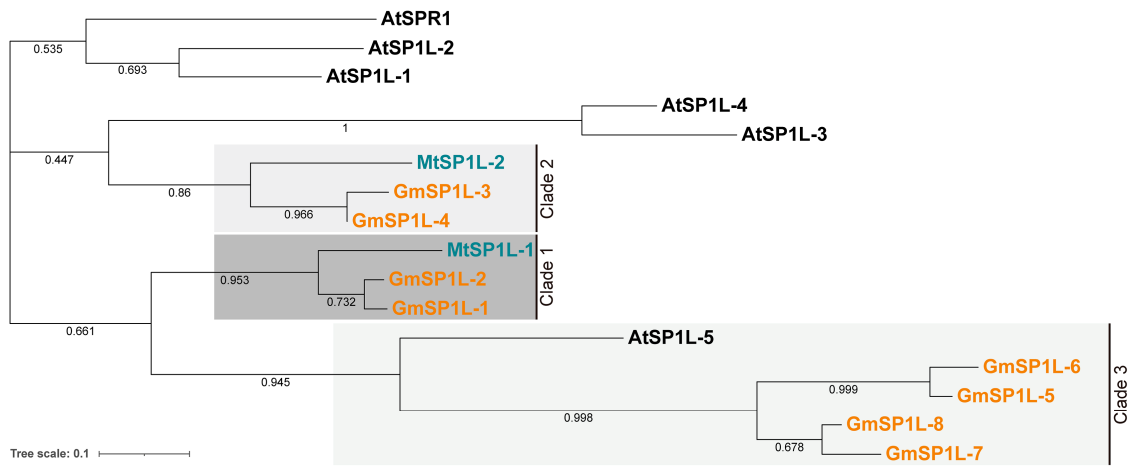

Supplement: Supplementary file 1 [file ijms-24-03958-s001.zip › Figure S1.pdf]

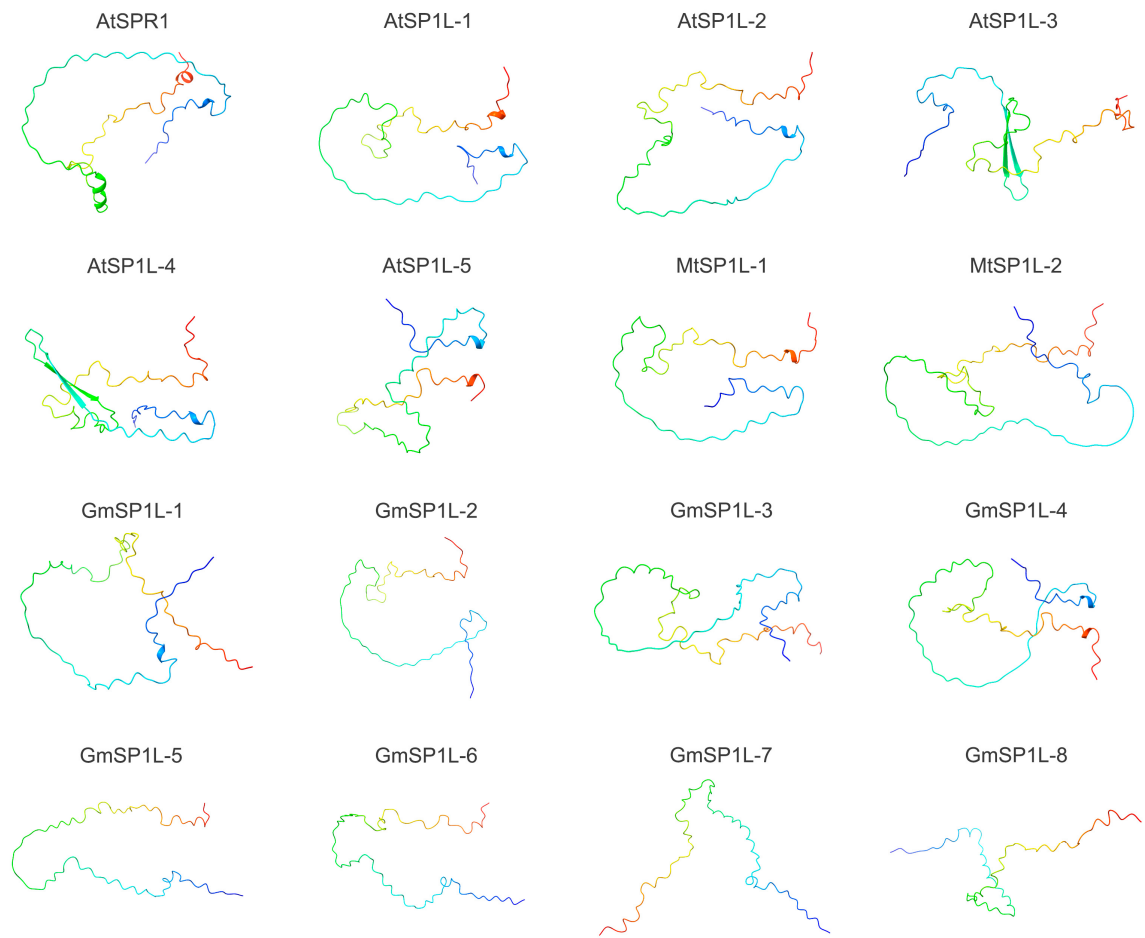

Supplement: Supplementary file 1 [file ijms-24-03958-s001.zip › Figure S2.pdf]

Motif 1

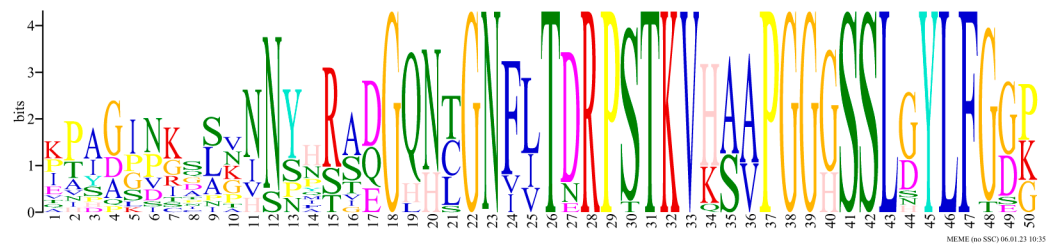

Motif 2

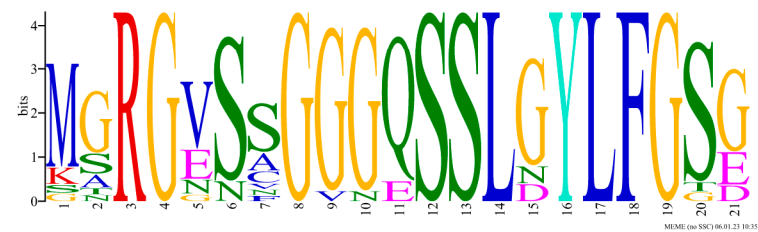

Motif 3

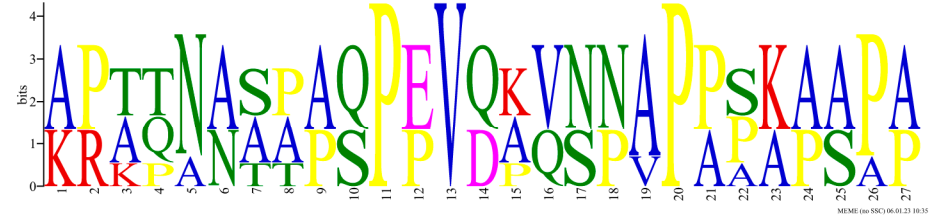

Motif 4

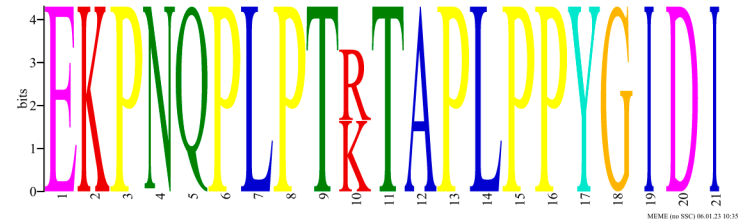

Supplement: Supplementary file 1 [file ijms-24-03958-s001.zip › Figure S3.pdf]
